# Comparison of the electrophysiological properties of the pulmonary veins between paroxysmal and persistent atrial fibrillation

**DOI:** 10.1002/joa3.12981

**Published:** 2023-12-29

**Authors:** Hitoshi Mori, Akira Hamabe, Daisuke Kawano, Tsukasa Naganuma, Mai Tahara, Yodo Gatate, Toyokazu Kimura, Hirotsugu Tabata, Ritsushi Kato

**Affiliations:** ^1^ Department of Cardiology Japan Self Defense Forces Central Hospital Tokyo Japan; ^2^ Department of Cardiology Saitama Medical University, International Medical Center Hidaka Japan

**Keywords:** atrial fibrillation, electrophysiological study, low‐voltage areas, pulmonary vein, substrate

## Abstract

**Background:**

The role of the pulmonary veins (PVs) as triggers in atrial fibrillation (AF) is well‐known; however, their detailed electrophysiological properties have not been thoroughly examined.

**Objective:**

This study aimed to investigate the electrophysiological properties of the PVs between paroxysmal AF (pAF) and persistent AF (perAF).

**Methods:**

Prior to catheter ablation in patients with pAF (*n* = 51) and perAF (*n* = 41), a voltage map of the left atrium and PVs was created under sinus rhythm, and the area of the myocardial sleeves in the PVs and their electrophysiological characteristics, including the pacing threshold and effective refractory period (ERP), were compared between the two groups.

**Results:**

Compared with perAF, the myocardial sleeves of PVs for pAF were significantly larger for all PVs. The ERP for perAF was significantly shorter than that for pAF for all PVs. The pacing threshold for perAF was significantly higher than that for pAF for the right and left superior PVs.

**Conclusion:**

In patients with perAF, a decrease in the normal myocardial sleeves and a shortening of the ERP were observed for all PVs. Those changes in the electrophysiological properties of the PVs might be related to the persistence of AF.

## Graphical abstract

In patients with persistent atrial fibrillation (AF), a decrease in the normal myocardial sleeves and a shortening of the effective refractory period were observed for all pulmonary veins (PVs). Those changes in the electrophysiological properties of the PVs might be related to the persistence of AF.

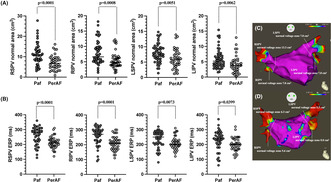

## 1 INTRODUCTION

Atrial fibrillation (AF) is caused by ectopic beats from the pulmonary veins (PVs), and pulmonary vein isolation (PVI) has become the cornerstone therapeutic strategy for the treatment of AF.^1^, ^2^, ^3^ PVI plays an important role in eliminating the triggers originating from the PVs. There is less of the AF substrate in paroxysmal AF (pAF) in the left atrial (LA) body, and a PVI alone is a sufficient treatment for pAF. A previous report noted that the AF‐free survival after a PVI for pAF is 64.1%–65.4%.^4^ On the other hand, persistent AF (perAF) has an arrhythmia driver that maintains the AF; hence, ablation of perAF is more challenging and is associated with less favorable outcomes.^5^ Substrate modification procedures, such as linear ablation of the left atrium^6^, ^7^ and complex fractionated potential ablation (CFAE),^8^ have been reported to be useful for reducing the recurrence of perAF. However, the STAR AF II trial demonstrated that there were no significant differences in the recurrence of AF among a PVI alone, PVI plus a line ablation, and a PVI plus a CFAE ablation.^9^ The recurrence rate of AF in the STAR AF II trial was 41% in the PVI alone group. However, the PRAISE AF trial, which investigated the efficiency of a PVI alone for perAF, revealed that 80% of the patients were free from arrhythmias at 12 months with a PVI alone.^10^ In that study, they enrolled patients who underwent a repeat procedure, regardless of any AF recurrence, and all reconnection sites were re‐ablated without any additional ablation, finally revealing an 80% arrhythmia freedom rate. That meant that a durable PVI is useful for treating perAF. We hypothesized that the PVs play not only a role as an AF trigger but also as an AF substrate. However, their detailed electrophysiological properties have not been thoroughly examined. The aim of this study was to investigate the electrophysiological properties of the PVs between pAF and perAF.

## 2 METHODS

### 2.1 Study subjects

**FIGURE 1:**
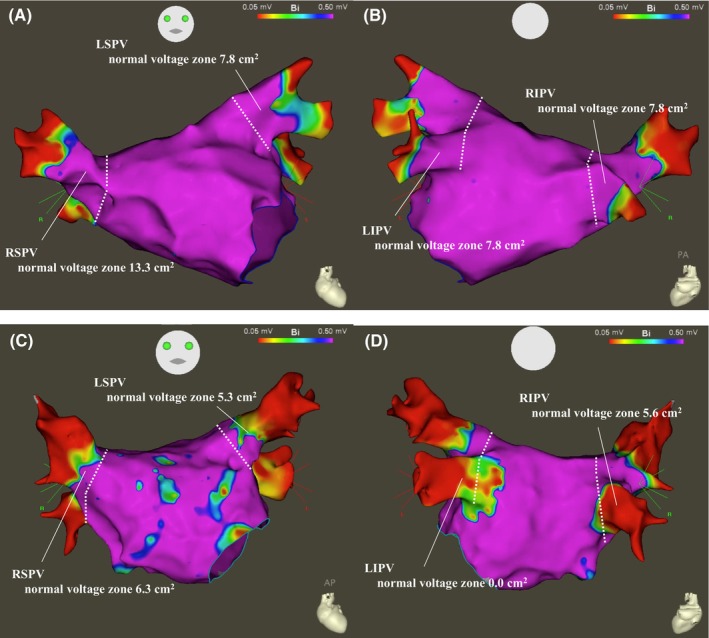
Voltage mapping in the left atrium and PVs. A voltage map of the LA and PVs was created using a five‐spline multi‐electrode catheter. The normal voltage of the PVs was defined as that of more than 0.5 mV. A perpendicular line was drawn from the carina to each PV, and the normal sleeve zone of the PV was measured distal to this line. The normal voltage areas for pAF (A, B) and perAF (C, D) were as follows: LSPV 7.8 cm^2^, RSPV 13.3 cm^2^, LIPV 7.8 cm^2^, and RIPV 7.8 cm^2^ (A, B) and LSPV 5.3 cm^2^, RSPV 6.3 cm^2^, LIPV 0.0 cm^2^, and RIPV 5.6 cm^2^ (C, D). LA, left atrial; LIPV, left inferior pulmonary vein; LSPV, left superior pulmonary vein; pAF, paroxysmal atrial fibrillation; perAF, persistent atrial fibrillation; PV, pulmonary vein; RIPV, right inferior pulmonary vein; RSPV, right superior pulmonary vein.

One hundred fifty‐eight AF patients who underwent a first AF ablation were enrolled in this study. All antiarrhythmic agents were discontinued for at least five half‐lives prior to the ablation. After the approach to the LA, we performed an electrical cardioversion to recover sinus rhythm in cases in which AF persisted. Before the PVI, all patients underwent voltage mapping of the LA and PVs under right atrial pacing (Figure 1). After the creation of a voltage map, an electrophysiological study (EPS) of each PV was performed to investigate the effective refractory period (ERP) and pacing threshold of the PVs. Patients whose AF was sustained after cardioversion were excluded from this study. Sixty‐six patients were excluded from this analysis, and the remaining 92 were enrolled in this study. The EPS findings were compared between the pAF group and perAF group. Since age affects the electrophysiological findings of the PVs, a comparison was also performed among 48 cases that were matched for age.

This study was performed in accordance with the provisions of the Declaration of Helsinki and local regulations. The research protocol was approved by the hospital's institutional review board (02‐006).

### 2.2 Ablation procedure

All ablation procedures were performed under deep sedation using propofol, dexmedetomidine, and pentazocine. The bispectral index was monitored and maintained at 40–60. After the voltage mapping and EPS, a PVI was performed by RF ablation or cryoballoon (CB) ablation. In the case of RF ablation, ablation was performed with a 3.5‐mm tip irrigation catheter (Thermocool SMARTTOUCH®; Biosense Webster, Irvine, CA, USA). CB ablation was performed with the 28‐mm second‐generation CB (Arctic Front Advance™; Medtronic, Minneapolis, MN, USA). After the completion of the PVI, a CTI ablation was performed in cases in which atrial flutter was recorded. Afterward, induction of any non‐PV origins was carried out using high‐dose isoproterenol, and if induced, ablation of the nonPV foci was additionally performed.

### 2.3 Voltage mapping of pulmonary veins

All procedures were performed under the guidance of a three‐dimensional mapping system (CARTO3®; Biosense Webster, Irvine, CA, USA), regardless of whether it was an RF or CB ablation. The voltage map was created before the PVI under right atrial pacing (cycle length = 600 ms) using a five‐spline multi‐electrode catheter (PentaRay, Biosense Webster, Irvine, CA, USA). A voltage map was created of the distal branch of the PV where no atrial potentials were recorded. A normal voltage of the PV was defined as more than 0.5 mV. A perpendicular line was drawn from the carina to each PV, and the normal sleeve zone of the PV was measured distal to this line (Figure 1). The normal sleeve zone was compared between the pAF and perAF groups.

### 2.4 Electrophysiological study of the pulmonary veins

After creating the voltage map, the ERP and pacing threshold of each PV were investigated using a PentaRay catheter in the proximal normal voltage area of the PVs, based on the voltage map. The ERP was investigated with extra‐stimulation at 600–300 ms and continued down every 10 ms. AF was induced after the extra‐stimulation in some patients. Once the AF was induced, the AF was terminated by external cardioversion. After returning to sinus rhythm, the extra‐stimulation was started at 600–100 ms and continued up every 10 ms. The ERP and pacing threshold were also compared between the pAF and perAF groups.

### 2.5 Statistical analyses

The statistical analyses were performed using JMP (versionPro16, SAS institute, Cary, North Carolina) and GraphPad Prism9 (GraphPad Software Inc., San Diego, CA) software. Continuous data are expressed as the mean ± SD for parametric data or median (interquartile range) for nonparametric data. The continuous variables were compared using a *t*‐test for parametric data and the Mann–Whitney test for nonparametric data. The categorical data were compared by a chi‐square test. A value of *p* < .05 was considered statistically significant.

## 3 RESULTS

### 3.1 Patient characteristics

Table 1 shows the baseline characteristics of the two groups, the pre‐matched (*n* = 92) and post‐matched (*n* = 48) groups. The LA diameter, prevalence of congestive heart failure, and BNP level were significantly higher in the perAF group, while the LVEF was significantly higher in the pAF group. These trends were also observed even after matching for age.

**TABLE 1:** Patient characteristics.

	Pre‐matched (*n* = 92)			Post‐matched (*n* = 48)		
	perAF (*n* = 41)	pAF (*n* = 51)	*p*	perAF (*n* = 24)	pAF (*n* = 24)	*p*
Clinical parameters						
Age	64.7 (±13.4)	51.6 (±11.9)	.2425	61.5 (±11.7)	61.5 (±11.7)	.99
BMI, kg/m^2^	24.8 (±3.8)	24.3 (±3.0)	.4873	25.1 (±4.0)	25.1 (±2.6)	.97
Gender, male, *n* (%)	31 (75.6)	39 (75.0)	.9461	21 (87.5)	20 (83.3)	.6821
Hypertension, *n* (%)	15 (36.6)	20 (38.5)	.8529	7 (29.2)	12 (50.0)	.1382
Diabetes, *n* (%)	3 (7.3)	6 (11.5)	.4942	2 (8.3)	3 (12.5)	.6355
Stroke, TIA, *n* (%)	3 (7.3)	2 (3.9)	.4612	3 (12.5)	1 (4.2)	.2862
CHF, *n* (%)	12 (29.8)	5 (9.6)	.0149*	6 (25.0)	2 (8.33)	.1143
CHADS2 score	1 (0–2)	0.5 (0–1)	.1400	1 (0–2)	1 (0–1)	.9036
Echocardiographic findings						
LA diameter, mm	45.8 (±8.5)	39.1 (±6.9)	<.0001*	45.7 (±8.6)	40.4 (±7.7)	.0300*
LVDd, mm	46.4 (±5.1)	46.3 (±5.9)	.9006	46.9 (±4.9)	45.3 (±4.7)	.2685
LVEF, %	62.0 (±9.7)	68.2 (±7.8)	.0012*	62.6 (±8.2)	68.9 (±7.2)	.0088*
Laboratory findings						
Cr, mg/dL	1.00 (±0.25)	1.03 (±1.20)	.8701	0.99 (±0.17)	1.26 (±1.74)	.4436
BNP, pg/mL	116.9 (68.1–167.4)	20.8 (8.4–51.8)	<.0001*	97.5 (53.5–129)	12.3 (7.6–41.0)	.0005*

*Note*: The continuous variables are shown as the mean ± SD and categorical variables as the number (%).

Abbreviations: AF, atrial fibrillation; CHF, congestive heart failure; LVDd, left ventricular diastolic diameter; LVEF, left ventricular ejection fraction; pAF, paroxysmal atrial fibrillation; perAF, persistent atrial fibrillation; TIA, transient ischaemic attack.

* *p* < .05.

### 3.2 Comparison of the PV sleeve areas

**FIGURE 2:**
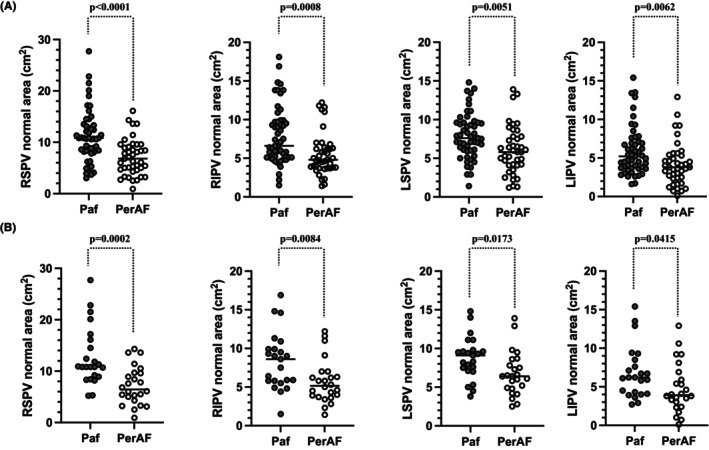
Comparison of the normal voltage areas between pAF and perAF. The comparison of the normal PV sleeve area in the pre‐matched group (A) and post‐matched group (B) between pAF and perAF. The normal PV sleeve area for pAF was significantly greater than that for perAF for all PVs. pAF, paroxysmal atrial fibrillation; perAF, persistent atrial fibrillation; PV, pulmonary vein.

Figure 2A shows the comparison of the normal PV sleeve area in the pre‐matched group between pAF and perAF. The normal PV sleeve area of pAF was significantly greater than that of perAF for all PVs (pAF vs. perAF; right superior pulmonary vein [RSPV], 11.1 cm^2^ [±5.1 cm^2^] vs. 7.1 cm^2^ [±3.5 cm^2^], *p* < .0001; right inferior pulmonary vein [RIPV], 8.0 cm^2^ [±3.8 cm^2^] vs. 5.5 cm^2^ [±2.8 cm^2^], *p* = .0008; left superior pulmonary vein [LSPV], 7.9 cm^2^ [±3.0 cm^2^] vs. 6.1 cm^2^ [±3.1 cm^2^], *p* = .0051; left inferior pulmonary veins [LIPV], 5.9 cm^2^ [±3.2 cm^2^] vs. 4.1 cm^2^ [±2.8 cm^2^], *p* = .0062).

Figure 2B shows the comparison of the normal PV sleeve area in the post‐matched group between pAF and perAF. The normal PV sleeve area for pAF was also significantly greater than that for perAF for all PVs (pAF vs. perAF; RSPV, 12.8 cm^2^ [±5.7 cm^2^] vs. 7.2 cm^2^ [±3.7 cm^2^], *p* = .0002; RIPV, 8.3 cm^2^ [±3.7 cm^2^] vs. 5.7 cm^2^ [±2.8 cm^2^], *p* = .0084; LSPV, 8.7 cm^2^ [±2.8 cm^2^] vs. 6.7 cm^2^ [±2.8 cm^2^], *p* = .0173; LIPV, 6.8 cm^2^ [±3.4 cm^2^] vs. 4.8 cm^2^ [±3.2 cm^2^], *p* = .0415).

### 3.3 Comparison of the pacing threshold between pAF and perAF

**FIGURE 3:**
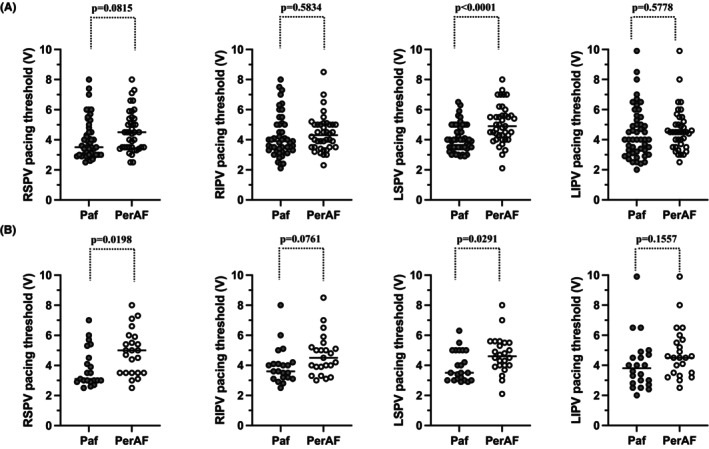
Comparison of the pacing threshold between pAF and perAF. The comparison of the pacing threshold in the pre‐matched group (A) and post‐matched group (B) between pAF and perAF. Significant differences were observed for the LSPV in the pre‐matched group and LSPV/RSPV in the post‐matched group. LSPV, left superior pulmonary vein; pAF, paroxysmal atrial fibrillation; perAF, persistent atrial fibrillation; PV, pulmonary vein; RSPV, right superior pulmonary vein.

Figure 3A shows the comparison of the pacing threshold in the pre‐matched group between pAF and perAF. The pacing threshold of the LSPV in the pAF group was significantly lower than that in the perAF group (pAF vs. perAF; RSPV, 4.0 V [±1.3 V] vs. 4.6 V [±1.3 V], *p* = .0815; RIPV, 4.3 V [±1.4 V] vs. 4.4 V [±1.2 V], *p* = .5834; LSPV, 4.1 V [±0.9 V] vs. 5.1 V [±1.3 V], *p* < .0001; left inferior pulmonary veins [LIPV], 4.5 V [±1.7 V] vs. 4.6 V [±1.4 V], *p* = .5778).

Figure 3B shows the comparison of the pacing threshold in the post‐matched group between pAF and perAF. The pacing threshold of the LSPV/RSPV in the pAF group was significantly lower than that in the perAF (pAF vs. perAF; RSPV, 3.8 V [±1.3 V] vs. 4.9 V [±1.5 V], *p* = .0198; RIPV, 4.0 V [±1.2 V] vs. 4.7 V [±1.4 V], *p* = .0761; LSPV, 4.0 V [±1.0 V] vs. 4.7 V [±1.2 V], *p* = .0291; LIPV, 4.1 V [±1.8 V] vs. 4.8 V [±1.7 V], *p* = .1557).

### 3.4 Comparison of the ERP between pAF and perAF

**FIGURE 4:**
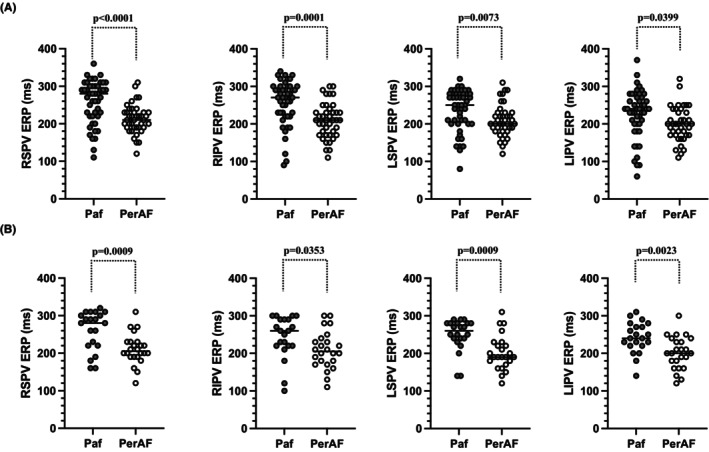
Comparison of the ERP between pAF and perAF. The comparison of the ERP in the pre‐matched group (A) and post‐matched group (B) between pAF and perAF. The ERP for pAF was significantly longer than that for perAF for all PVs. ERP, effective refractory period;pAF, paroxysmal atrial fibrillation; perAF, persistent atrial fibrillation; PV, pulmonary vein.

Figure 4A shows the comparison of the ERP in the pre‐matched group between pAF and perAF. The ERP for pAF was significantly longer than that for perAF for all PVs (pAF vs. perAF; RSPV, 259.0 ms [±57.8 ms] vs. 212.7 ms [±38.4 ms], *p* < .0001; RIPV, 256.4 ms [±60.0 ms] vs. 209.5 ms [±47.3 ms], *p* = .0001; LSPV, 237.0 ms [±54.4 ms] vs. 208.3 ms [±42.9 ms], *p* = .0073; LIPV, 225.4 ms [±65.6 ms] vs. 199.8 ms [±45.8 ms], *p* = .0399).

Figure 4B shows the comparison of the ERP in the post‐matched group between pAF and perAF. The ERP for pAF was significantly longer than that for perAF for all PVs (pAF vs. perAF; RSPV, 260.5 ms [±52.8 ms] vs. 210.8 ms [±40.6 ms], *p* = .0009; RIPV, 243.3 ms [±56.7 ms] vs. 208.8 ms [±50.1 ms], *p* = .0353; LSPV, 249.1 ms [±43.0 ms] vs. 202.1 ms [±46.2 ms], *p* = .0009; LIPV, 242.4 ms [±41.5 ms] vs. 201.3 ms [±43.5 ms], *p* = .0023).

## 4 DISCUSSION

### 4.1 Major findings

The major findings in our study were as follows: (1) The normal voltage areas of the PVs in the pAF cases were significantly greater than that in the perAF cases. (2) Compared to the pAF cases, the pacing threshold tended to be higher in the perAF cases. (3) The ERP of the PVs was longer in all PVs for pAF.

### 4.2 Electrical remodeling of the PVs for perAF

Previous studies have reported that low‐voltage areas are more frequently observed in the LA body in perAF cases, and a high recurrence rate of AF after catheter ablation is noted in those cases.^11^ Furthermore, the bipolar voltages in several parts of LA were significantly lower in patients with perAF than in those with pAF.^12^ It is believed that those low voltages represent atrial fibrosis and electrical remodeling, indicating slow conduction and contributing to the substrate of AF.^13^ However, there have been no reports comparing the low‐voltage areas in the PVs between pAF and perAF. Our study revealed that in pAF cases, the myocardial sleeves in the PVs tended to be longer, whereas in the perAF cases, those myocardial sleeves tended to be shorter, which might have been related to the electrical remodeling of the PVs.

After the age‐matched analysis, the pacing threshold in the RSPV and LSPV was significantly higher in the perAF cases than in pAF cases. Although no statistical significance was observed, the pacing threshold in the RIPV and LIPV also tended to be higher in the perAF cases. Previous reports about ventricles noted that a higher pacing threshold could have an increased risk of arrhythmia occurrence.^14^ Similarly, the increase in the pacing threshold in the PVs due to electrical remodeling would contribute to the arrhythmogenesis in perAF.

Furthermore, the ERP of the atrial muscle is shortened in patients with AF, which contributes to the maintenance of AF.^15^ The ERP of the PVs was longer in the pAF cases for all PVs, as compared to that in the perAF cases. The shorter PV myocardial sleeves in the perAF cases suggested that there was electrical remodeling, which may have caused the shortened ERP. Our study suggested the possibility that a short ERP of the PVs might contribute to the substrate of perAF and persistence of AF.

### 4.3 Pulmonary vein isolation as a substrate ablation of persistent AF

In the STAR AF2 trial, the AF‐free survival rate with a PVI alone was reported to be 52%, whereas in the PRAISE study, the AF‐free survival with a PVI alone was reported to be 80%.^9^, ^10^ The common cause of arrhythmia recurrence after ablation was reconnections of the PVs, and the durability of the PVI contributed to this higher AF‐free survival in the PRAISE study. Originally, a PVI was considered useful for isolating ectopic beats from PVs, i.e., the origin of AF.^1^ However, the fact that the sinus rhythm could be obtained in 80% of cases with perAF by performing a highly durable PVI suggests the possibility that the PVs may function as a substrate to some extent. Our study suggested that the shortening of the ERP and the increase in the pacing threshold in the perAF cases may have contributed to the persistence of AF, suggesting that a PVI may serve not only as a trigger ablation but also as a substrate ablation of AF.

### 4.4 Study limitations

Our study had several limitations. First, this was a single‐center study and the study population was relatively small. A larger cohort study is needed to evaluate our results. Second, the ERP of the PVs varies depending on the pacing site in the PVs.^16^ In our study, the ERP was compared using pacing from a single site in a high‐voltage area, and the results might have differed if performed as an average of pacing from multiple sites in the PVs. Finally, in cases of perAF, cardioversion was performed just before the EPS. Therefore, there was a possibility that the short period of time from the cessation of AF may have affected the results of the electrophysiological tests.

## 5 CONCLUSIONS

In patients with perAF, a decrease in the normal myocardial sleeves, an increase in the pacing threshold, and a shortening of the ERP were observed in the PVs. The PVs may serve not only as triggers but also as substrates of AF. Therefore, a durable PVI plays an important role in the treatment of perAF.

## AUTHOR CONTRIBUTIONS

Hitoshi Mori, study conception and design; Akira Hamabe, Daisuke Kawano, Tsukasa Naganuma, Mai Tahara, Yodo Gatate, and Toyokazu Kimura, data collection and data analysis; Hirotsugu Tabata and Ritsushi Kato, manuscript revision and study supervision.

## CONFLICT OF INTEREST STATEMENT

HM received lecture fees from Biosense Webster Japan and Boston Scientific Japan. HM, DK, NT, and RK received grant support from Boston Scientific Japan and Abbott Medical Japan.

## ETHICS STATEMENT

The study protocol was approved by the hospital's institutional review board (IRB number; 02‐006).

## PATIENT CONSENT STATEMENT

Patient consent has been obtained in an opt‐out manner.

## CLINICAL TRIAL REGISTRATION

Not applicable.
